# Contribution to characterization of skin field cancerization activity: morphometric, chromatin texture, proliferation, and apoptosis aspects^☆^^☆☆^

**DOI:** 10.1016/j.abd.2019.03.003

**Published:** 2019-10-25

**Authors:** Anna Carolina Miola, Mariana Anteghini Castilho, Juliano Vilaverde Schmitt, Mariangela Esther Alencar Marques, Helio Amante Miot

**Affiliations:** a Department of Dermatology and Radiotherapy, Faculdade de Medicina de Botucatu, Universidade Estadual Paulista, Botucatu, SP, Brazil; b Department of Dermatology, Instituto Lauro de Souza Lima, Bauru, SP, Brazil; c Discipline of Radiology and Diagnostic Imaging, Faculdade de Medicina de Botucatu, Universidade Estadual Paulista, Botucatu, SP, Brazil; d Department of Pathology, Faculdade de Medicina de Botucatu, Universidade Estadual Paulista, Botucatu, SP, Brazil

**Keywords:** Carcinoma, Squamous cell, Keratosis, Actinic, Skin neoplasms

## Abstract

**Background:**

A skin field cancerization is a cutaneous area with subclinical changes resultant from chronic sun exposure, with a higher predisposition to development of pre-neoplastic and neoplastic lesions. So far, there are no well-defined objective parameters that can indicate their degree of activity.

**Objectives:**

To describe and compare morphometric aspects and expression of factors related to apoptosis and cell proliferation in actinic keratosis (AK), in both photoexposed and photoprotected epidermis.

**Methods:**

A cross-sectional study of patients with actinic keratosis in the forearms, biopsied at two points: the actinic keratosis and the axillary region. The biopsies of the actinic keratosis, perilesional area, and axilla were evaluated through keratinocyte intraepithelial neoplasia (KIN), and immunohistochemistry of p53, survivin, and Ki67. Nuclear morphometry of basal layer cells was performed through digital image analysis: entropy, area, perimeter, Ra, fractal dimension, circularity, color intensity, and largest diameter.

**Results:**

There were 13 patients included and 38 actinic keratosis biopsied. In morphometry, 1039 nuclei were analyzed, of which 228 represented axillary skin, 396 demonstrated actinic keratosis, and 415 represented the perilesional area to the actinic keratosis. There was a significant difference (*p* < 0.05) in all variables tested for the topographies evaluated. A significant correlation was identified between nucellar morphometric elements, KIN, proliferation markers, and apoptosis. Joint patterns of p53, Ki67, and KIN discriminated the topographies sampled.

**Study limitations:**

This was a cross-sectional study with a small number of patients.

**Conclusions:**

There are patterns of proliferation, resistance to apoptosis, and different cellular morphometrics between photoprotected skin and photoexposed skin. The joint expression of p53, Ki67, and KIN can characterize skin field cancerization activity.

## Introduction

The concept of field cancerization was proposed by Slaughter in 1953 and comprises a seemingly normal integument area, but with subclinical and multifocal alterations, composed of cells genetically altered as a result of chronic sun exposure.^1^, ^2^ The concept of skin field cancerization (SFC) suggests that the clinically normal skin adjacent to actinic keratoses (AKs) is the focus of clonal expansion of genetically altered cells, which would explain the occurrence of new AKs or other cutaneous neoplasms in the same area, in addition to local recurrence of tumors considered completely excised by histopathological analysis.^3^

Recently, SFC has been studied due to its clinical importance; its stabilization can prevent the appearance of neoplasias, and the recurrence or evolution of existing lesions.

Currently, AKs, which are pre-neoplastic epithelial lesions, are considered markers of SFC activity.^4^ However, so far, there are no well-defined objective parameters that can indicate their degree of activity.

Determining the extent and intensity of the activity of an SFC is important in order to create diverse and adequate treatment and prevention strategies for each type of patient, as well as to evaluate the response and prognosis after the establishment of appropriate therapies.^5^ Survivin, p53, and Ki67 are nuclear markers present in proliferative cells, and are expressed in neoplastic and pre-neoplastic lesions, which may aid in the characterization of an active SFC.^6^

Synthesis of p53 induces DNA repair and apoptosis. Mutations in p53 are the most common genetic alterations in human neoplasms.^7^ Recently, the analysis of exons in the p53 gene in tumors of the aerodigestive tract has demonstrated a probable molecular method for the detection of the cancerization field.^8^ In addition, mutations in p53 can be found in AKs and squamous cell carcinomas (SCCs) in most cases, and in higher concentrations in SCC, whereas patients without suspected cancer lesions do not express p53 mutations, suggesting that mutations in p53 may be involved in the conversion of AK to SCC, and, consequently, may indicate activity in the field of cutaneous cancer.^9^

The Ki67 antigen is a marker of cell proliferation^10^, ^11^ correlated with tumor growth and higher risk of metastasis.^12^, ^13^ The nuclear expression of Ki67 has been previously studied in cutaneous neoplasms and is evidenced in AK, Bowen's disease, basal cell carcinoma (BCC), and SCC.^14^, ^15^ To date, there are no studies describing the expression of Ki67 in SFC activity.

Survivin is a protein expressed by proliferative keratinocytes and can be found in the cytoplasm or nucleus.^16^ It acts in regulation of the cell cycle and in control of apoptosis. Its function varies according to the intracellular location^17^: while nuclear expression is more associated with cell division and is essential for mitosis, the presence of survivin in the cytoplasm is considered cytoprotective.^18^ A high level of nuclear survivin is predictive of greater malignancy or worse prognosis when analyzed in epithelial tumors.^19^, ^20^, ^21^, ^22^ Moreover, the location of nuclear survivin in the epidermis layers also influences and indicates prognosis: while the presence of survivin in the basal layer can be found in unaltered epithelium, the expression of nuclear survivin in the suprabasal layers indicates rupture of epidermal cell homeostasis and consequently, mitotic activity, which may aid in the characterization of SFC.^23^

Nuclear morphological analysis can provide clues about cellular physiology and contribute to the diagnostic and prognostic evaluation of neoplastic lesions. In addition, changes in nuclear chromatin architecture may indicate intense mitotic activity and correlate with neoplastic proliferation rates. Thus, morphometry and nuclear texture characteristics have been studied as prognostic factors in many neoplasms.^24^, ^25^, ^26^, ^27^ Investigations on nuclear morphometry and chromatin heterogeneity have been performed on BCCs; however, there are no studies characterizing such parameters in SFC and correlating them with field activity.^28^

The use of morphometric and immunohistochemical techniques, together with clinical and histopathological data, can provide information on the biological behavior and contribute to the evaluation of treatment effectiveness.

## Methods

A cross-sectional study was conducted with adult patients of both genders, with AK in the forearms, biopsied at two points: the skin comprising an AK and another point of the axillary skin. This study was approved by the institution's ethics committee and all patients enrolled signed an informed consent.

The biopsies of the AK, the perilesional area, and the photoprotected area were analyzed by histopathology (HE) using the keratinocyte intraepithelial neoplasia (KIN) method. KIN classification is a way of classifying the intensity of keratinocyte atypia by its presence in the epidermal levels, so that KIN I presents focal atypia in the lower third of the keratinocytes; KIN II shows depolarization in the prickly-granulosa layers; and KIN III shows depolarization in all epidermal layers.^29^ For purposes of statistical analysis, normal axillary skin was considered as KIN 0.

For immunohistochemistry, the percentage of cells stained by survivin, p53, and Ki67 in the hotspots of the AK, perilesional, and axillary areas were analyzed.

**Figure 1:**
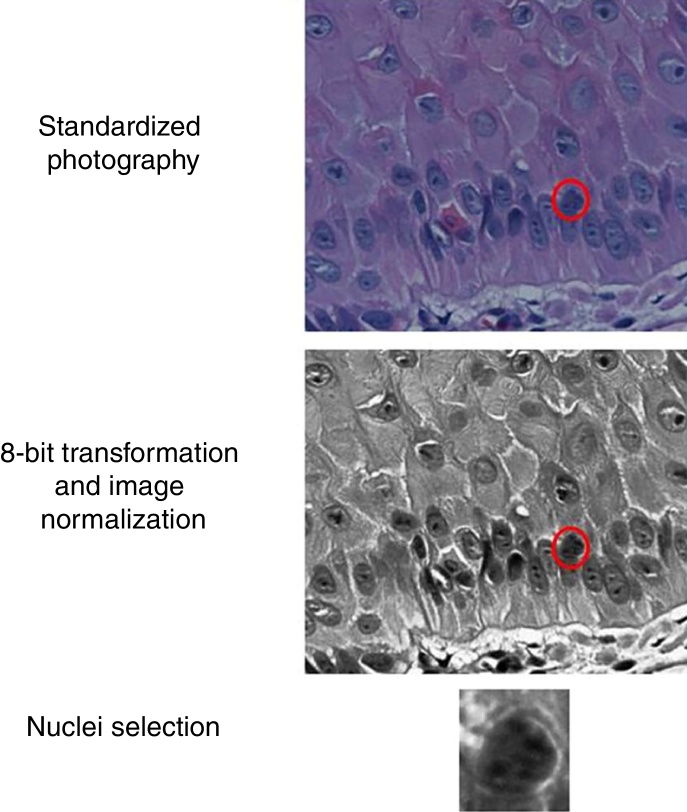
Representative scheme of image preparation and nuclei selection.

The slides were photographed in high resolution (400×) using a Coolscope II microscope (Nikon Instruments Inc. – Tokyo, Japan, 2009), with about 30 nuclei/field. The basal layer epidermal nuclei were sliced manually, and their images were transformed to eight bits and normalized. ImageJ software was used (Fig. 1). The resulting images were submitted to the extraction of entropy, color intensity, fractal dimension, Ra, circularity, perimeter, and area indices.

Regarding the statistical analysis, data were compared between the topographies by the Jonckheere–Terpstra test. Normality was estimated by the Shapiro–Wilk test.^30^ The correlations between the indicators were estimated by the Spearman coefficient and presented as a thermal map.^31^ The behaviors of the variables were compared by multiple correspondence analysis and arranged by a perceptual map.^32^ Values of *p* < 0.05 were considered significant.

## Results

This study included 13 patients: four women and nine men, from 37 to 88 years old, in whom 38 AKs were sampled.

In the morphometry, 1039 nuclei were analyzed, of which 228 represented axillary whole skin, 396 were from AKs, and 415 were from the perilesional areas of the AKs. The mean morphometric values are shown in Table 1.

**Table 1:** Values of the variables according to the location of the skin sample. All variables resulted in *p* < 0.05.

Nuclear parameters^a^	Axilla	Perilesional	AK
Area	1,278.30 (1,128.0–1,266.62)	1,481.57 (1,312.56–2,070.25)	1,823.58 (420.54–2,295.45)
Perimeter	140.98 (135.64–145.06)	157.52 (148.81–209.99)	178.52 (152.27–213.58)
Circulation	0.81 (0.75–0.84)	0.74 (0.71–0.77)	0.73 (0.69–0.78)
Larger diameter	51.69 (48.96–52.45)	56.62 (54.15–69.30)	52.11 (54.17–74.93)
Median of color	33.94 (30.22–35.78)	39.19 (31.59–43.38)	43.64 (36.12–50.24)
Entropy	5.30 (5.17–5.32)	5.26 (5.18–5.35)	5.34 (5.26–5.54)
Ra	1,346.55 (988.80–2,026.91)	2,181.88 (1,601.59–7,777.78)	3,579.69 (1,744.43–8,115.73)
Fractal dimension	2.45 (2.44–2.45)	2.46 (2.45–2.48)	2.47 (2.45–2.50)

a Median (p25–75).

**Figure 2:**
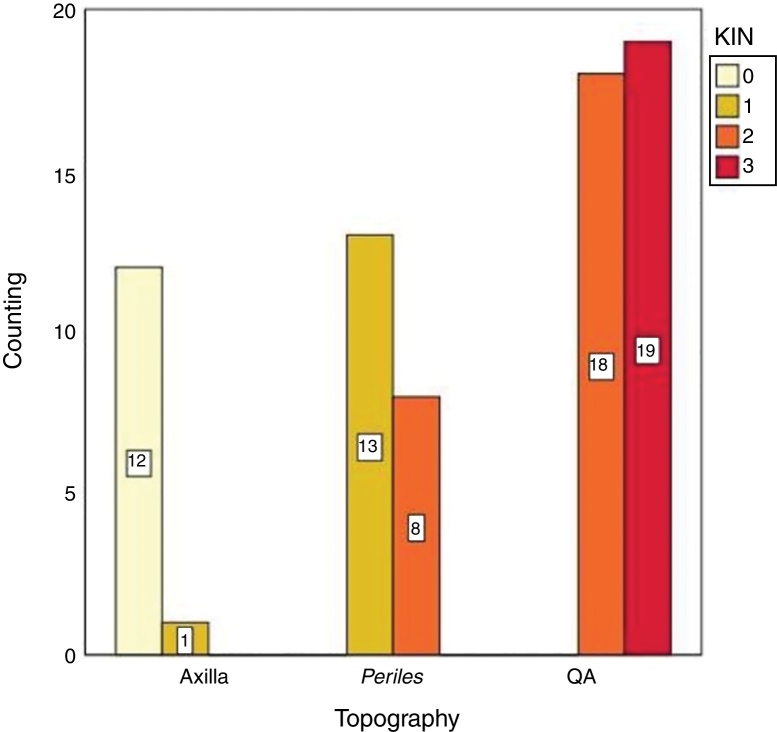
Distribution of the keratinocyte intraepithelial neoplasia (KIN) evaluation according to topography.

There was a significant difference (*p* < 0.05) in the values of photoprotected and photoexposed skin in all tested variables (p53, Ki67), as observed in Table 2. In addition, KIN was directly related to photoexposure, tending toward zero in the photoprotected skin (Fig. 2).

**Table 2:** Percentage of cells expressing survivin, p53, and Ki67 in the AK, photoexposed skin, and photoprotected skin. All variables resulted in *p* < 0.05.

Markers^a^	Axilla	Perilesional	AK
Ki67	18.68 (14.29–26.62)	23.98 (17.59–29.46)	42.07 (35.03–51.61)
Suprabasal survivin	11.58 (6.71–15.50)	18.57 (10.06–28.43)	38.16 (23.22–46.41)
p53	13.81 (5.38–20.12)	23.88 (16.83–37.78)	65.35 (54.64–79.61)

a Median (p25–75).

**Figure 3:**
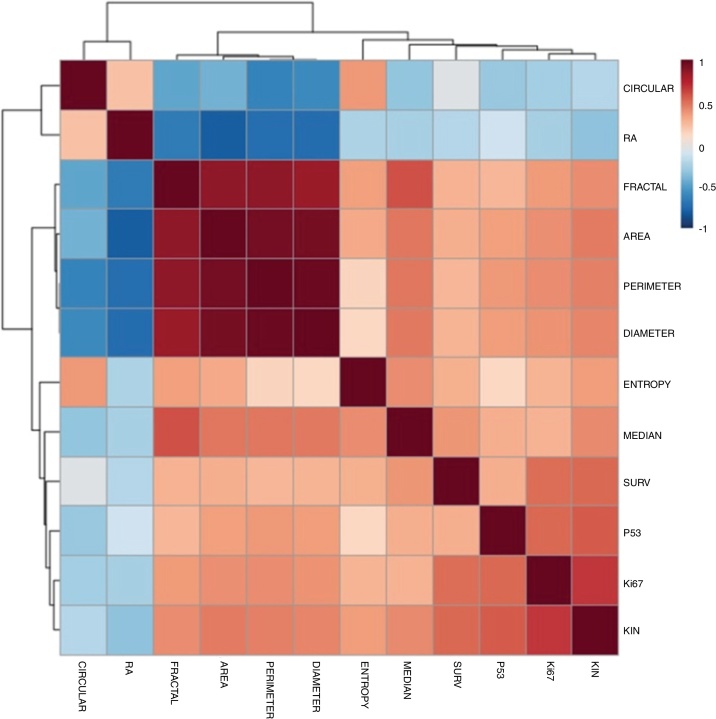
Thermal map of the correlations (Spearman) between the morphometric and apoptosis-related indicators and proliferation. All correlations resulted in *p* < 0.05.

Correlation between morphometric, proliferation, and apoptosis indicators was identified (Fig. 3). The elements that most correlated with KIN were p53 and Ki67. There was an intense correlation between the morphometric elements.

**Figure 4:**
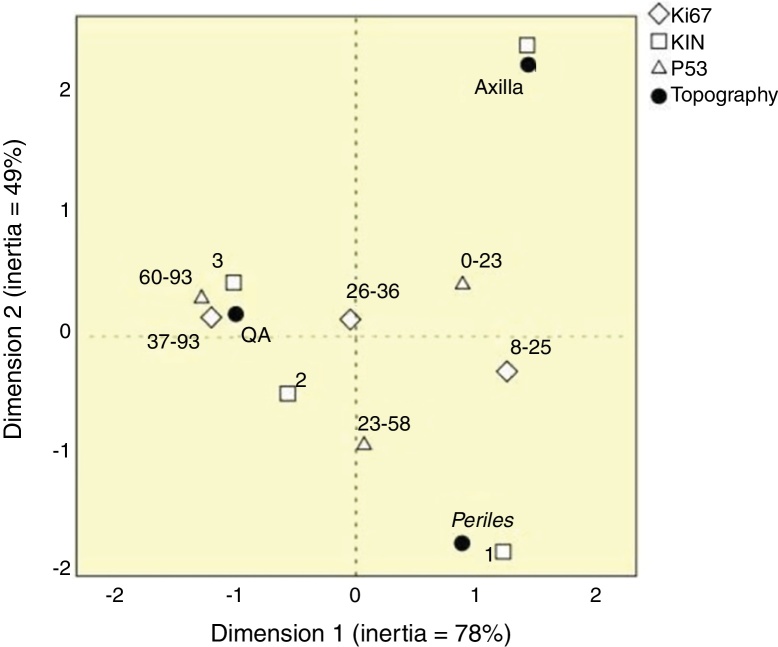
Perceptual map of the distribution of p53, Ki67, and keratinocyte intraepithelial neoplasia (KIN) according to topography.

None of the variables tested was highlighted as an individual marker of SFC activity; however, KIN, p53, and Ki67 together could characterize the behavior of topographies (Fig. 4). There was great spatial proximity of AK with KIN 3, and the highest values of Ki67 and p53; the exposed skin had close proximity to KIN 1, and intermediate values of the other markers. However, the axillary epidermis had greater proximity to KIN 0.

## Discussion

The SFC, in this group of samples, was characterized by increased epithelial proliferation, increased expression of apoptosis-related factors, and alterations in nuclear morphology, in agreement with the literature.^9^, ^11^ There was a progressive expression of these variables in protected skin in relation to the sun-exposed skin and AKs, showing that chronic exposure to ultraviolet radiation promotes tissue changes in the SFC, with correlation between the expression of these factors and the degree of histological dysplasia of the epithelium.

The associations of epithelia with the expression of KIN, p53, and Ki67 changed proportionally according to sun exposure, which suggests them as strong candidates for the characterization of the SFC, and indicates that the analysis of their activity can be used in therapeutic clinical trials and in the development of measures aimed at preventing the development of skin cancers.

There was also an increase in the expression of suprabasal survivin, with a relation directly proportional to the increase of solar exposure and presence of AA, as expected in the literature^23^; however, its correlation with Ki67 and p53, which are well established markers,^11^, ^12^, ^13^, ^14^ was less intense (Graph 2). In addition, further correlation studies are needed between the expression of suprabasal survivin and SFC activity so that there is a higher level of evidence for these findings.

Alterations in nuclear morphometry and chromatin texture are used in pathology as criteria for differentiation between healthy tissues and neoplasias.^24^, ^31^ In addition, tissues under stress, such as ultraviolet radiation, may also present nuclear alterations, usually related to the intensity of metabolic activity.^24^, ^28^ In this study, phenotypically, the genomic damage to the SFC manifested as changes in the shape of the nucleus and heterogeneity of chromatin, correlated among themselves and with progressive changes according to photoexposure and the presence of AKs, supporting the evidence for genomic instability and suggesting that these alterations occur in a simultaneous intranuclear manner.

The main limitations of the study are the small number of participants, although they allowed a sufficient number of lesions for the analysis. As the study is transversal, although AK and SFC are unstable entities, a longitudinal design could provide more consistent elements of their activity linked to the oncological prognosis.^33^, ^34^

The histological characterization of SFC should provide additional contributions to the AK count, quality of life scales, and clinical severity scores^4^, ^35^, ^36^ in understanding the underlying phenomena of SFC. These findings are being tested in clinical trials of SFC treatments such as photodynamic therapy,^37^ ingenol mebutate, and 5-fluorouracil, and according to a scale of severity of AKs in development for the present authors’ SFC study group.

## Conclusion

Morphometric and chromatin texture variables showed differences in comparison of photoexposed and photoprotected skin. KIN, p53, and Ki67 – together – allowed the characterization of SFC.

## Financial support

None declared.

## Author's contribution

Anna Carolina Miola: Approval of the final version of the manuscript; elaboration and writing of the manuscript; obtaining, analyzing and interpreting the data; critical review of the literature.

Mariana Anteghini Castilho: Approval of the final version of the manuscript; conception and planning of the study; obtaining, analyzing and interpreting the data.

Juliano Vilaverde Schmitt: Approval of the final version of the manuscript; obtaining, analyzing and interpreting the data; effective participation in research orientation; intellectual participation in propaedeutic and/or therapeutic conduct of the cases studied; critical review of the literature; critical review of the manuscript.

Mariangela Esther Alencar Marques: Approval of the final version of the manuscript; critical review of the manuscript.

Helio Amante Miot: Statistical analysis; approval of the final version of the manuscript; effective participation in research orientation; intellectual participation in propaedeutic and/or therapeutic conduct of the cases studied; critical review of the literature.

## Conflicts of interest

None declared.
